# Treatment of Prion Disease with Heterologous Prion Proteins

**DOI:** 10.1371/journal.pone.0131993

**Published:** 2015-07-02

**Authors:** Pamela J. Skinner, Hyeon O. Kim, Damani Bryant, Nikilyn J. Kinzel, Cavan Reilly, Suzette A. Priola, Anne E. Ward, Patricia A. Goodman, Katherine Olson, Davis M. Seelig

**Affiliations:** 1 University of Minnesota, Veterinary and Biomedical Sciences Department, Saint Paul, MN 55108, United States of America; 2 University of Minnesota, Veterinary Clinical Sciences Department, Saint Paul, MN 55108, United States of America; 3 University of Minnesota, School of Public Health, Division of Biostatistics, Minneapolis, MN, 55455, United States of America; 4 Laboratory of Persistent Viral Diseases, National Institute of Allergy and Infectious Diseases, Rocky Mountain Laboratories, Hamilton, Montana 59840, United States of America; Deutsches Zentrum für Neurodegenerative Erkrankungen e.V., GERMANY

## Abstract

Prion diseases such as Creutzfeldt-Jakob disease in humans, bovine spongiform encephalopathy in cattle, and scrapie in sheep are fatal neurodegenerative diseases for which there is no effective treatment. The pathology of these diseases involves the conversion of a protease sensitive form of the cellular prion protein (PrP^C^) into a protease resistant infectious form (PrP^sc^ or PrP^res^). Both *in vitro* (cell culture and cell free conversion assays) and *in vivo* (animal) studies have demonstrated the strong dependence of this conversion process on protein sequence homology between the initial prion inoculum and the host’s own cellular prion protein. The presence of non-homologous (heterologous) proteins is often inhibitory to this conversion process. We hypothesize that the presence of heterologous prion proteins from one species might therefore constitute an effective treatment for prion disease in another species. To test this hypothesis, we infected mice intracerebrally with murine adapted RML-Chandler scrapie and treated them with heterologous prion protein (purified bacterially expressed recombinant hamster prion protein) or vehicle alone. Treated animals demonstrated reduced disease associated pathology, decreased accumulation of protease-resistant disease-associated prion protein, with delayed onset of clinical symptoms and motor deficits. This was concomitant with significantly increased survival times relative to mock-treated animals. These results provide proof of principle that recombinant hamster prion proteins can effectively and safely inhibit prion disease in mice, and suggest that hamster or other non-human prion proteins may be a viable treatment for prion diseases in humans.

## Introduction

Prion diseases, also known as transmissible spongiform encephalopathies (TSE), are rare progressive neurodegenerative diseases that are transmissible between species [1–3]. These diseases include Creutzfeldt-Jakob disease (CJD) in humans; bovine spongiform encephalopathy (BSE) in cattle [4]; chronic wasting disease (CWD) in deer and elk [5]; and scrapie in sheep, goats, and experimentally infected rodents [1]. Prion diseases belong to a growing family of disorders that are attributed to misfolding and aggregation of proteins, including Alzheimer’s disease, Parkinson’s disease and systemic amyloidosis [6,7].

Some distinguishing features of prion disease are their wide phenotypic variety and their multiple methods of acquisition (sporadic, genetic or acquired) [8]. The infectious agent in these diseases are prions (proteinaceous infectious particles) [9]. Prion diseases are believed to involve misfolding of an endogenous cellular prion protein, PrP^C^, into a variant self-replicating isoform, PrP^res^ [10]. The mechanism of this is uncertain, but it is believed that an aggregate of PrP^res^ protein binds the cellular PrP^C^ and catalyzes its conversion to an infectious form [11]. The misfolding and accumulation of prion proteins is thought to be the basis of prion disease pathogenesis and infectivity [2,12–14].

The mouse Prnp gene encodes a 254 amino acid long prion protein, which is post-translationally processed to an approximately 210 amino acid long protein via cleavage at both its N and C terminus [15–17]. Structural studies suggest that it is arranged with a disordered amino-terminal tail and a globular C-terminal domain composed of three α-helices and two anti-parallel β-sheets [18,19]. It is anchored to the outer cell surface membrane via a glycosylphosphatidylinositol (GPI) anchor which helps tether the protein to the outer cell surface membrane [20]. Whereas PrP^C^ exists predominately as a monomer/dimer in an alpha helical configuration, the variant PrP^res^ is aggregated in nature and exists predominately in a β-pleated sheet rich conformation [11,21]. This aggregated misfolded PrP^res^ state is characterized by resistance to protease degradation and chemical disinfection [22]. It is proposed that the normal replication of PrP^res^ is dependent on recruitment of PrP^C^ into this altered PrP^res^ configuration following a nucleation-dependent polymerization mechanism [23].

The primary structure of host PrP^C^ is a major determinant of prion disease susceptibility. Transgenic mice that lack PrP^C^ are resistance to prion infection [24]. A high degree of sequence identity between the infecting prion and the host PrP^C^ is often necessary for efficient prion replication [25]. Moreover, differences in the PrP^C^ sequence have been proposed to be involved in resistance to cross species infection (species barriers) and prion strains [26–28]. However, this effect is not strictly dependent on amino acid homology and appears to be more dependent on subtle structural variations, most notably differences within the loop/turn structures [26,29]. Experiments in transgenic mice, tissue culture cells and cell-free systems have identified the middle third region of the prion protein as being important for the autocatalytic conversion process [30]. Polymorphisms in this middle third region of the protein can confer resistance to prion disease, whereas homology at critical amino acid residues has been demonstrated to facilitate cross-species transmission of prion disease. Transgenic mice expressing a variant mouse PrP containing three amino acid substitutions in the β2-α2 loop completely resisted infection with two different strains of prions [31].

There is currently no effective treatment for prion diseases in humans, and these diseases in humans are always fatal. Drugs have been identified which show some efficacy in treating prion diseases in tissue culture systems or in whole animal systems [32]. Two of these compounds, quinacrine and pentosan polysulfate have been used as compassionate therapy of patients with CJD or vCJD, however, no therapeutic benefit was seen [33,34]. Other treatment strategies for prion diseases have been attempted including vaccination and immunotherapy, but these strategies have had limited success [35]. Another recent therapeutic target for prion disease is the unfolded protein response since misfolding of prion proteins during disease development stimulates the activation of the unfolding response pathway. Chemical inhibition of this pathway has been demonstrated to be neuroprotective and to abrogate disease development in prion-infected mice [36]. Another recent treatment strategy has employed lentivirus vectors expressing silencing RNAs directed against the cellular form of the prion protein [37]. These lentivirus vectors were used to transduce mouse embryonic stem cells and create chimeric mice expressing various levels of the silencing RNAs. Scrapie survival times were extended in those mice that were highly chimeric for the transgene and that showed reduced PrP^C^ expression in the brain.

We hypothesize that heterologous prion proteins can be used as a viable treatment for prion diseases. In other words, normal prion proteins from one species can be used to treat prion disease in another species. The rationale for this hypothesis comes from several previously published studies. Induced expression of hamster prions in scrapie-infected mouse cells almost completely eliminates the accumulation of the misfolded prion protein isoform (PrP^res^) in contrast to cell lines not expressing the hamster gene [38]. This suggests that heterologous hamster prion proteins may actually inhibit PrP^res^ production in the scrapie-infected cells. Similarly, scrapie-infected mouse cells that are induced to express a rabbit prion gene produce substantially less PrP^res^ as compared to mouse cells that do not express rabbit prion proteins [26], suggesting that rabbit prion proteins may interfere with mouse PrP^res^ formation.

Inhibition of PrP^res^ formation by heterologous prion proteins has also been observed in studies of transgenic mice. In transgenic mice expressing both endogenous mouse prion proteins as well as an exogenous hamster prion gene, onset of disease and death after mouse scrapie infection is significantly delayed compared to wild-type mice [39]. Furthermore, transgenic mice expressing human PrP^C^ are resistant to infection with human prions [40], but become susceptible upon ablation of the mouse Prnp gene [41] indicating that the mouse prion proteins inhibit the propagation of human prions. The goal of this study was to test the proof of principle that heterologous prion proteins can be used to effectively treat prion disease.

Scrapie infection in mice has been well-studied [42] and intracerebral inoculation of mice with low doses of scrapie inoculum offers a model of iatrogenic prion disease transmission that occurs in humans. The mouse scrapie model system in conjunction with recombinant hamster prion proteins offers a relatively safe and effective system in which to test the hypothesis that heterologous prion proteins can be used to effectively treat prion diseases. For this study we evaluated the effectiveness of treating scrapie-infected mice with bacterially expressed and purified recombinant hamster prion proteins. The results of this study provide proof of principal that heterologous prion proteins can be used safely and effectively to treat prion diseases.

## Materials and Methods

### Preparation of recombinant hamster PrP proteins

Recombinant hamster PrP 23-231(HaPrP) in a pET plasmid [43] was used to transform *E*. *coli* BL21(DE3) cells. The protein was recovered using the BugBuster kit (Novocastra) and affinity purified over at NiNTA Superflow resin (Qiagen) as previously described [43]. The protein solution was diluted to 150 mL with H_2_O, sterile filtered, and then dialyzed (MWCO 10,000) against 2 L of 10mM sodium acetate pH 5 at 4°C. The protein solution was then sterile filtered and stored at -80°C. Centriprep 10 concentrators (Millipore) were used to concentrate the protein prior to use.

### Treatment of scrapie-infected mice

Four week-old C57BL/6 female mice were separated into four groups with 13 animals in each treatment group (high dose, low dose and mock) and 10 animals in the untreated uninfected group. Recombinant hamster prion proteins (HaPrP) or vehicle alone were administered both at the time of inoculation with of scrapie and orally the following day. Mice were anaesthetized with isoflurane and intracerebrally inoculated with 5 μl of a 0.01% RML-Chandler strain scrapie brain homogenate (obtained from mice with symptomatic scrapie infection) diluted in PBS containing 2% fetal calf serum, plus 45ul of either HaPrP (0.7mg/ml) for high dose treatment, HaPrP (0.35 mg/ml) for low dose treatment, or 45 ul of vehicle only (10 mM sodium acetate, pH 5) for mock-treated mice. The intracerebral inoculations with the HaPrP were delivered together in one injection. The titer of the scrapie inoculum used is estimated to be at least as high as the stock of RML-Chandler used to derive this inoculum, which was 2X10^8^ infectious units/gram of brain [44]. The following day, mice were treated orally with 100ul of recombinant proteins or vehicle alone for mock-treated mice. Archived tissues from age matched animals that were mock-infected with 1% normal mouse brain homogenate were included as additional negative controls. Animals were evaluated daily following infection, weekly during the first months, and then daily in later months for signs of scrapie-related symptoms including ataxic gait, hind limb paresis, decreased motility, dull eyes, flattened stature, weight loss, and kyphosis. At the same time, animals were also evaluated for signs of non-scrapie related disease or discomfort. Animals with symptoms of scrapie, or otherwise found to be suffering, as determined by the overseeing veterinarian, were euthanized with CO_2_. At experimental endpoints mice were similarly euthanized with CO_2_. Three of the 16 aged study mice were euthanized due to the development of severe dermatitis. Two were in the high dose-treated group and sacrificed on the last day of the study (452 days post-infection). The other was in the low dose treated group, and sacrificed at 347 days post-infection, towards the end of the study. The development of severe dermatitis is common in C57BL/6 mice [45] and not thought to be attributed to the scrapie infection or our HaPrP treatment.

### Ethics Statement

The University of Minnesota has an approved Animal Welfare Assurance #A3456-01 on file with the NIH Office of Laboratory Animal Welfare and complies with the USDA Animal Welfare Act Regulations, and the Public Health Service Policy on Humane Care and Use of Laboratory Animals. All animal studies at both the University of Minnesota and the Rocky Mountain Laboratories were carried out in strict accordance with the recommendations in the Guide for the Care and Use of Laboratory Animals of the National Institutes of Health. The Academic Health Center and the Rocky Mountain Laboratories are fully accredited by the Association for the Assessment and Accreditation of Laboratory Animal Care, International. The University of Minnesota Institutional Animals Care and Use Committee (IACUC) approved animal protocol (#0702A03021) and the Rocky Mountain Laboratories Animal Care and Use Committee approved Protocol 03–06 for use in the study.

### Hanging wire assay

An adaptation of the hanging wire assay [46] was used as a simple and atraumatic assessment of neuromuscular function during the latter portion of the study. Mice used their forelimbs or hindlimbs in any combination to grasp a wire mesh suspended 20 cm above a cage bottom filled with wood shavings. A series of three 120-second trials was conducted on each test day and the times until mice dropped were averaged.

### Western blot analysis

To detect PrP^res^ protein accumulation in the spleen and brain, 20% solutions of spleen and brain tissue were prepared in 0.01M Tris-HCL pH 7.4 and 0.005 M MgCl, were treated with 10mg/ml DNase (Roche) and 0.25mg/ml collagenase (Sigma), and sonicated. Tissue lysates were mixed with an equal volume (1ml) of 20% sarcosyl (Sigma) and 3 ml of 10% sarcosyl in 0.01 M Tris HCl pH 7.4 and spun in an ultracentrifuge for thirty minutes in a Beckman ultracentrifuge rotor Type 70.1 (Beckman Coulter) at 5000 RPM. Supernatant was transferred to a new tube and centrifuged at 55,000 RPM for two hours. The resultant pellet was resuspended in 1 ml water, sonicated, and then treated with 100μg/ml proteinase K (Fisher scientific) for 1 hour at 37°C. Reactions were terminated with 3 mM PMSF (Sigma). Samples were again spun at 55000 rpm for one hour and pellets resuspended in 5X western blot sample buffer (Pierce), sonicated, and boiled prior to loading on gels.

For detection of PrP^C^ and GAPDH, brain and spleen tissue was lysed in RIPA buffer (50 mM Tris HCl pH 8, 150 mM NaCl, 1% NP-40, and 0.5% sodium deoxycholate, 0.1% SDS) containing protease inhibitors (Sigma) and centrifuged at 12000 rpm for 20 minutes. BCA protein assay kit (Thermo scientific) was used to determine protein concentrations of resultant supernatant. 6.25 mg/well of protein was run through pre-cast 4–12% polyacrylamide gels (Invitrogen) in Tris-glycine and transferred to PVDF membranes (Millipore). Blots were probed with mouse antibodies directed against PrP (SAF-83, Cayman chemical) and GAPDH (6C5, Millipore). Blots were scanned and the density of background subtracted bands determined using image J software. Relatively shorter exposure blots were used for densitometry to avoid saturated bands, and relatively longer exposure blots were used for presentation.

### Immunohistochemistry

Mice were perfused and tissues fixed overnight at 4°C with freshly made paraformaldehyde. Tissues were then dehydrated in alcohol and paraffin embedded. Four micron thick tissue sections were treated with antigen retrieval reagent (Diva Decloaker; Biocare, Concord, CA) and then blocked in blocking sniper solution (Biocare) for one hour, and then stained with GFAP antibodies (clone GA-5, Biomeda) diluted 1:1000 and counter stained with hematoxylin using MACH 3 or MACH 4 Polymer kit (Biocare) and the Vectastain ABC Kit (Vector Labs). Stained sections were scanned using an Aperio tissue scanner using a 20X objective. To determine relative amounts of GFAP DAB staining, Adobe Photoshop CS3 Extended software was used to determine mean gray values for tissue areas. The mean gray value obtained in a region with hematoxylin staining and little to no DAB staining was subtracted from this value to discriminate DAB staining from hematoxylin staining. Only areas of the brain included in all sections were included in quantitative analysis. Cerebellum, brainstem, and olfactory bulb were not included in every section and were excluded. Any areas of folded tissue or overt artifacts were manually removed from images prior to quantification.

In later experiments to identify and quantify reactive astrocytes and microglia, 5 μm-thick sections were immunostained using antibodies directed against GFAP and Iba1, respectively. Each slide was subjected to heat-induced epitope retrieval using Decloaking Chamber NxGen (Biocare Medical, Concord, CA) and a citrate (ph 9.0) buffer. Endogenous peroxidase activity was quenched with 3% H_2_O_2_ in methanol and nonspecific background was blocked using Background Punisher (Biocare Medical). Sections were incubated with either an anti-GFAP antibody (ab7260, Abcam, diluted 1:2500) or an anti-Iba1 antibody (ab105464, Abcam, diluted 1:100) for 2 hours at room temperature or overnight at 4-degrees C, respectively. Slides were then incubated with an HRP-conjugated secondary antibody (Mach 4; Biocare Medical), incubated with the AEC chromagen (Romulin AEC; Biocare Medical), and counterstained with hematoxylin. For quantification, seven areas (rostral cortex, striatum, thalamus, hypothalamus, hippocampus, cerebellum, and medulla) were evaluated by an observer that was blinded to the treatment group (CF). From each area 3–4, 200X magnification images were captured using an upright microscope and positive stained cells were manually quantified using ImageJ software. The average number of cells and standard error were calculated using Microsoft Excel.

### Spongiosis Scoring

Spongiosis scoring was performed and lesion profiles were generated using a modified variation of previously published criteria. For each lesion profile, the particular neuroanatomic regions were selected based on (1) the ease by which a particular section could be reproducibly identified and (2) correlation with previously published rodent scrapie studies. Using hematoxylin-and-eosin stained slides, the following sites were evaluated: (1) cerebral cortex dorsal to the septal nucleus, (2) cerebral cortex dorsal to the corpus callosum, (3) hippocampus, (4) thalamus, (5) hypothalamus, (6) cerebellar cortex, and (7) medulla. For each site, the severity of vacuolar lesions was graded 0 (no lesions) to 4 (extensive vacuolization) by a veterinary pathologist (DMS). To generate lesion profiles, mean scores for each area were calculated and plotted + SEM.

### Statistical Methods

To test for differences in survival time distributions between animal groups the Peto Peto modification of the Gehan-Wilcoxon test was used [47] (this test is one version of the log rank test). This test is suited to detecting early differences in the survival distribution in the presence of right censored data. There is right censoring in this data set as not all animals had been diagnosed with symptoms of scrapie at termination of the study and some were removed from the study for reasons unrelated to scrapie or the treatments. Such animals are used to compute the number of animals at risk of having an event prior to becoming right censored.

To test for differences in the temporal trajectories of performance on the wire hanging assay, a mixed model was used with indicator variables for group membership, a linear time trend, interactions between the group indicator variables and the time trend and animal specific random effects. The inclusion of random effects allows us to correctly model the dependence among observations from the same animal at different time points. These models were fit using restricted maximum likelihood and the Wald test associated with the slopes for the interaction between time and group membership. This provides a test for differences in the rate of decline in performance on this assay. One of the most important aspects of using mixed models in the context of longitudinal data analysis is that all of the data is used, and, in particular, specific time points are not selected by the data analyst (as this obviously biases the analysis). Since the data are necessarily values between 0 and 120, we first divide all of the data by 120 then use a logistic transformation to these observations to improve their approximation to normality.

The Wilcoxon rank sum was used to test for differences between 2 groups except for the astrocytosis analysis where the sample size was too small for this test to have any power to detect a significant difference. For that analysis, 2 sample *t*-tests were used on the logarithm of the levels to improve the approximation to normality.

All statistical calculations were conducted using the software R version 2.10.1. The R packages survival and nlme had the functions necessary for this analysis.

## Results

### Study Design

Since previous studies suggested that heterologous PrP proteins might be useful for treating prion disease, we undertook this proof of principal study. This study included a total of 39 mice that were divided into 3 treatment groups (high dose, low doseand mock) comprised of 13 mice each, as well as a group of 10 additional mice that were not infected or treated (Table 1). All of the mice in the 3 treatment groups were infected via intracerebral inoculation with 50ul of 0.001% RML-Chandler strain of scrapie brain homogenate. This was the lowest dose predicted to induce infection in 100% of the animals. The first group (high dose) was treated with a relatively high dose of 31.5 ug of bacterially expressed and purified recombinant hamster PrP amino acids 23–231 (HaPrP) via intracerebral injection coincident with the scrapie inoculation and orally with 70 ug of HaPrP the following day. The second group of mice was similarly treated with a lower dose of HaPrP equal to 1/2 the high dose, and the third control group were mock treated with vehicle alone. The reason we included the oral dose the next day was to 1) encourage immunological tolerance of HaPrP and 2) to perhaps add some additive inhibitory effect to PrP^res^ that was transmitted systemically in the blood after the intracerebral inoculation. Two animals (1 mock-treated and 1 low-dose-treated) did not survive intracerebral inoculation. This loss was anticipated as normally a small subset of animals does not survive intracerebral inoculation. Of the remaining 34 mice, a subset of mice were sacrificed relatively early in the course of infection at 108 days post-infection and examined for the development of disease associated pathology (3 mice per group) and for the development and accumulation of misfolded protease resistant prion proteins, PrP^res^ (4 mice per group). The remaining mice in the treatment groups (5–6 mice per group), as well as the 10 uninfected and untreated mice were left to age and were used to determine the onset of scrapie-associated symptoms and survival times.

**Table 1:** Distribution of mice in study.

Group			Pre-clinical 108 dpi		Clinical symptoms
	Total # mice	Died after injection	Histology	Prp^sc^ detection	Motor skills Symptoms Survival
Mock-treated	13	1	3	4	5
Low dose treated	13	1	3	4	5
High dose treated	13	0	3	4	6
Not infected or treated	10				10

### Reduced PrP^res^ accumulation in brain and spleen of high-dose-treated mice

**Fig 1:**
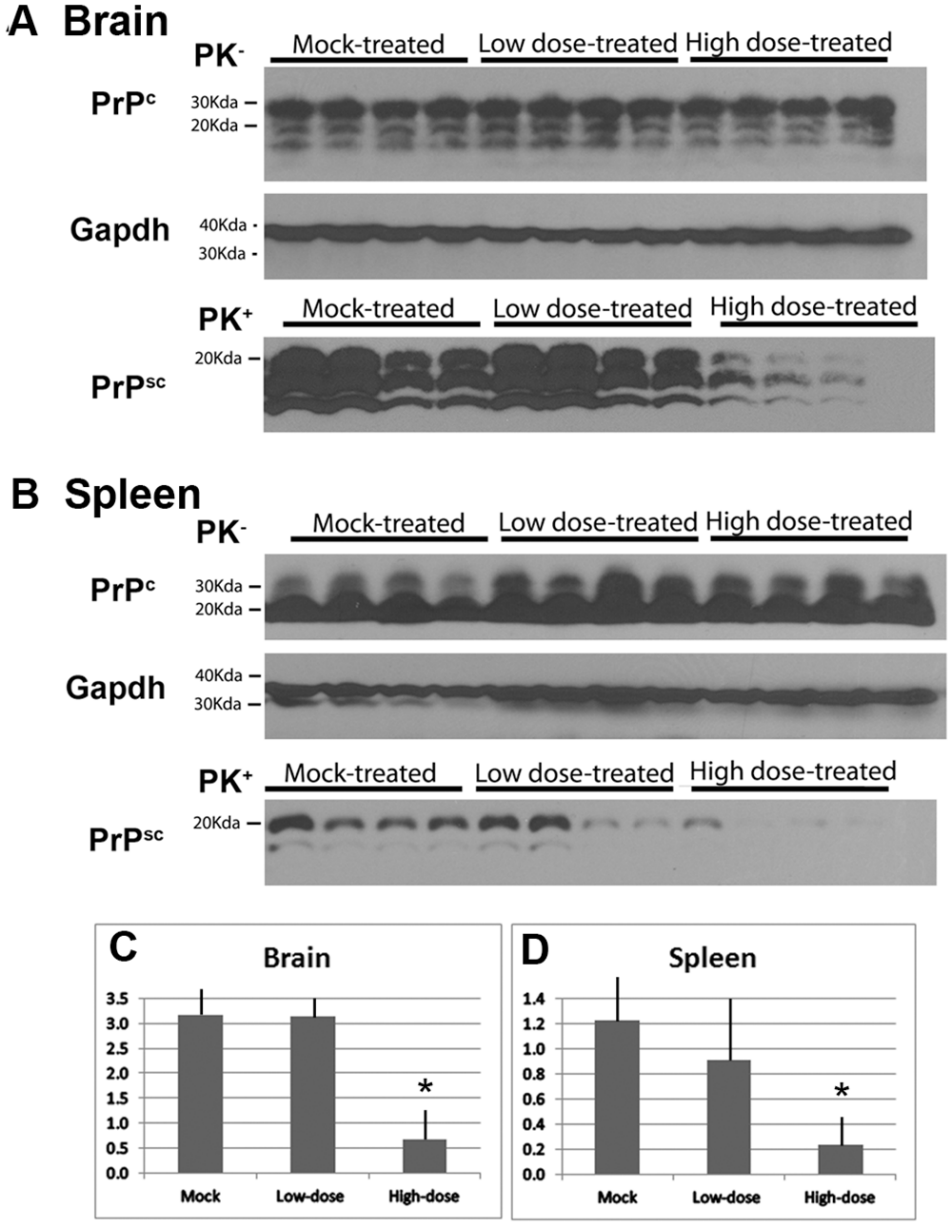
Reduced PrP^res^ accumulation in brain and spleen of high-dose-treated mice. Western blot analysis of prion protein from the brain (A) and spleen (B) from scrapie-infected mice that were mock-treated with vehicle alone, treated with a relatively low dose of hamster prion protein, or treated with a relatively high dose of hamster prion protein, collected at 108 days post-scrapie infection. Mouse antibodies directed against PrP (SAF83) were used to detect PrP^C^ and Proteinase K treated (PK) PrP^res^. GAPDH staining was used as a loading control. The average density of PrP^res^ bands for each group is shown for the brain (C) and spleen (D). Significantly lower levels of PrP^res^ were observed in the high-dose treated animals relative to mock-treated animals (*p* = 0.0286 by the Wilcoxon rank sum test, also, see Fig 3). There was no observed difference between the low dose and mock infected groups (*p* = 0.686 by the Wilcoxon rank sum test).

In order to examine the effect of our treatment on the pre-symptomatic mice (108 days post-infection), a subset of each treatment group was sacrificed. Western Blot analysis was used to evaluate the levels of both normal prion protein, PrP^C^, and of misfolded protease-resistant disease-associated prion protein, PrP^res^, in brain and spleen tissues. All of the mice showed detectable PrP^res^ indicating that all of the animals were infected and that treatment did not prevent infection or development of PrP^res^. Notably, there was significantly lower levels of PrP^res^ in the high-dose treated animals relative to mock-treated animals (*p* = 0.0286 by the Wilcoxon rank sum test, (Fig 1). Therefore the high dose treatment was able to effectively reduce the development of disease associated misfolded prion protein, although it did not prevent infection or the formation of PrP^res^. There was no observed difference between the low dose and mock infected groups (*p* = 0.686 by the Wilcoxon rank sum test).

### Decreased disease-associated pathology in brains of treated mice

**Fig 2:**
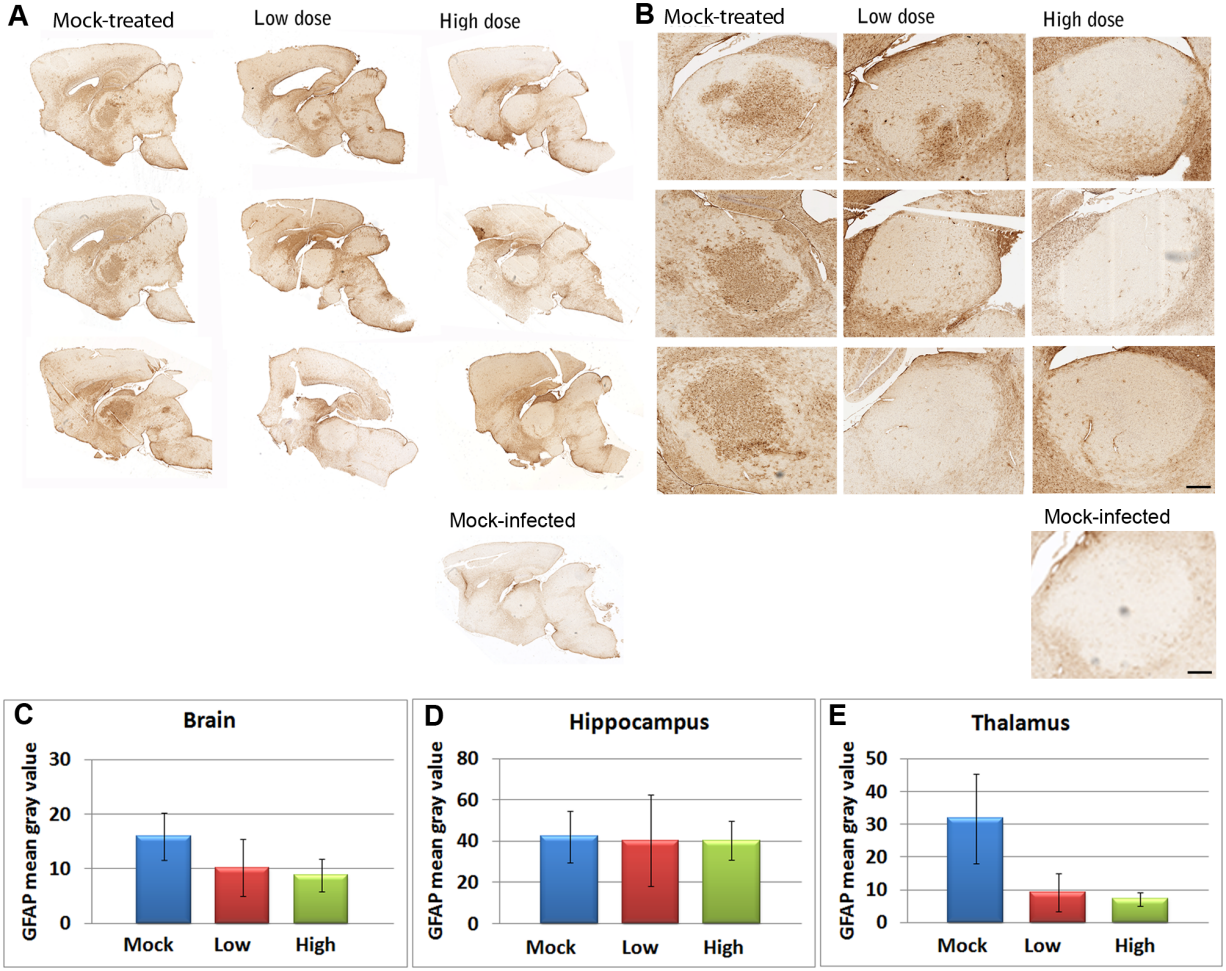
Decreased astrocytosis in brains of high-dose treated mice. Anti-GFAP antibody (brown) and hematoxylin (blue) staining in A) whole brain and B) enlargement showing thalamus. For comparison, similar staining is presented from an aged matched negative control animal that was mock-infected with a 1% homogenate of normal mouse brain. For this analysis, regions of brain not present in all sections were removed (including cerebellum, brainstem, and olfactory bulb). Scale bars = 200 μm. Levels of GFAP staining are shown for C) whole brain, D) hippocamus, and E) thalamus. The high-dose-treated mice showed a trend of less GFAP accumulation in the brain compared to mock-treated mice (*p* = 0.103 by a 2 sample *t*-test on the logarithms of the levels), whereas no difference was detected between the low-dose-treated and mock infected mice (*p* = 0.906 again by a 2 sample *t*-test). Decreases in high-dose treated verses mock-treated mice were most pronounced in the thalamus (*p* = 0.000557 by a 2 sample *t*-test); no significant differences in staining were seen amongst groups in the hippocampus (*p* = 0.825 for the high-dose group compared to the mock treated group and *p* = 0.839 for the low-dose group compared to the mock treated group, again with a 2 sample *t*-test).

In order to check the functional significance of decreased PrP^res^ in the treated animals, we next compared the brains of the treated and untreated animals by immunohistology. A major pathological change that occurs in the central nervous system during prion diseases is an increase in astrocyte number, a condition termed astrocytosis. Astrocytosis can be evaluated in mice by staining glial fibrillary acidic protein (GFAP), which is nearly exclusively expressed in astrocytes in the brain. Prior to the onset of clinical symptoms, at 108 days post-infection, we evaluated GFAP expression in the brains of treated and mock-treated mice to determine levels of astrocytosis. All of the mice showed evidence of astrocytosis (Fig 2). Representative images from an aged matched untreated mock-infected mouse are shown for comparison. Although all of the treated mice and mock-treated mice exhibited astrocytosis, quantification of GFAP levels in whole brain sections and regions of the brain showed differences amongst the groups. The high-dose-treated mice showed a trend of less GFAP accumulation in the brain compared to mock-treated mice (*p* = 0.103 by a 2 sample *t*-test on the logarithms of the levels), whereas no difference was detected between the low-dose-treated and mock infected mice (*p* = 0.906 again by a 2 sample *t*-test) as seen in Fig 2. Decreases in high-dose treated verses mock-treated mice were most pronounced and highly significant in the thalamus (*p* = 0.000557 by a 2 sample *t*-test); no significant differences in staining were seen amongst groups in the hippocampus (*p* = 0.825 for the high-dose group compared to the mock treated group and *p* = 0.839 for the low-dose group compared to the mock treated group, again with a 2 sample *t*-test). These results further indicate that all of the study mice became infected with scrapie and suggest that treatment with the relatively high-dose of HaPrP resulted in delayed development of disease associated pathology in scrapie infected mice.

In addition to an increases in astrocyte number, prion diseases are also characterized by neuronal vacuolation (spongiosis) and increases in microglial number. To further assess scrapie-associated pathological changes in the treated vs. mock-treated mice, we evaluated spongiosis lesion profiles, and levels of activated astrocytes and microglia in seven brain regions of treated and mock-treated mice. As seen in S1 Fig, the mock and low dose treated mice had a similar severity of spongiosis across 6 of 7 brain regions. However, the high dose treated mice had less severe spongiosis in 4 of the 7 areas in comparison to the mock and low dose treated animals. These areas included the cerebral cortex dorsal to the septal nucleus, the cerebral cortex dorsal to the corpus callosum, the thalamus, and hypothalamus. Astrocytosis was similar in all groups in all brain regions except the thalamus, in which the low- and high-dose-treated groups substantially lower levels. In order to determine whether there was an increase in microglial cell numbers, immunohistochemical studies with Iba1 antibodies, which stain microglia were performed. Interestingly, both the high- and low-dose-treated animals demonstrated substantially lower numbers of Iba1 positive microglia in all brain regions as compared to mock-treated animals (S1 Fig).

### Treatment delayed loss of motor function in scrapie-infected mice

**Fig 3:**
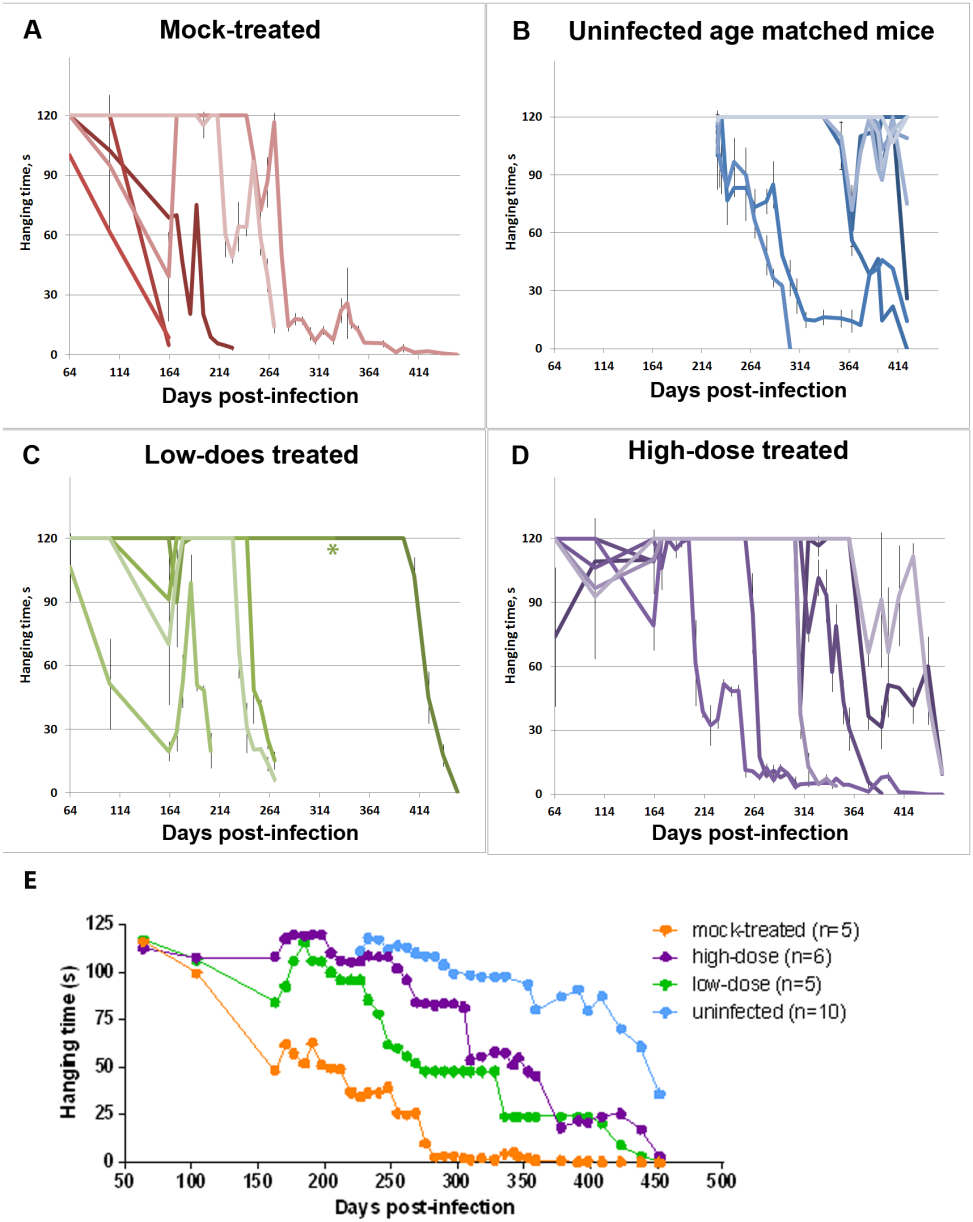
Treatment with heterologous recombinant HaPrP delayed loss of motor function. The hanging wire assay was used as an objective measure of motor coordination and muscle strength. The graphs show the results of A) mock-treated mice, B) uninfected age matched mice, C) low-dose-treated mice, and D) high-dose-treated mice. E) Shows the mean values for each group. The X-axis shows the day post-infection and the Y-axis shows the time of latency to fall from a wire grid in a 120 second trial. In A-D), individual averages of triplicate trials are shown with error bars showing the standard deviation. Mixed model analysis (which uses the data from all time points to test for differences between groups) finds significant differences between both treatment dose groups and the mock infected group (*p*<0.001 for both tests). Significant differences (p<0.001) were also seen between the three groups (mock, low dose, high dose) and the uninfected age matched mice. Note, one of the low-dose treated animals included in this study (indicated with an asterisk in C) was euthanized due to the development of a skin disease towards the end of the study at 347 dpi.

A wire-hanging assay was used as an objective measure of motor coordination and muscle strength in scrapie-infected mice treated with hamster prion protein relative to mock-treated mice. The wire-hanging assay measures the latency of mice to fall from a wire grid. For this study, mice used their forelimbs or hindlimbs in any combination to grasp a wire mesh suspended 20 cm from the counter. A series of three 120-second trials was conducted on each test day and the times until mice dropped were averaged. Testing was initiated at 64 days post-infection and continued until mice were sacrificed. The results of this assay showed that the high-dose-treated group of mice developed loss of motor function at substantially longer times post-infection than mock-treated mice, although significantly less than the uninfected control mice (Fig 3). The results from mixed model analysis of the data show strong support for differences between the groups. There was strong evidence of a more rapid decline in performance in the mock-treated mice than either the treated mice (both dose levels) and the uninfected control mice (*p*<0.001 for all three tests). These results demonstrate that the HaPrP treated mice maintained better motor coordination and strength over time relative to the mock-treated mice.

In addition, we found that mice began to perform poorly on the wire-hanging assay on average 3 weeks prior to their displaying observable scrapie-associated clinical symptoms based on cage behavior. These findings indicate that the wire-hanging assay was more sensitive than our observation of cage behavior in first detecting the clinical onset of prion disease in the study mice.

### Treatment with heterologous hamster prion protein delayed the onset of symptoms and prolonged survival of RML-Chandler scrapie infected mice

**Fig 4:**
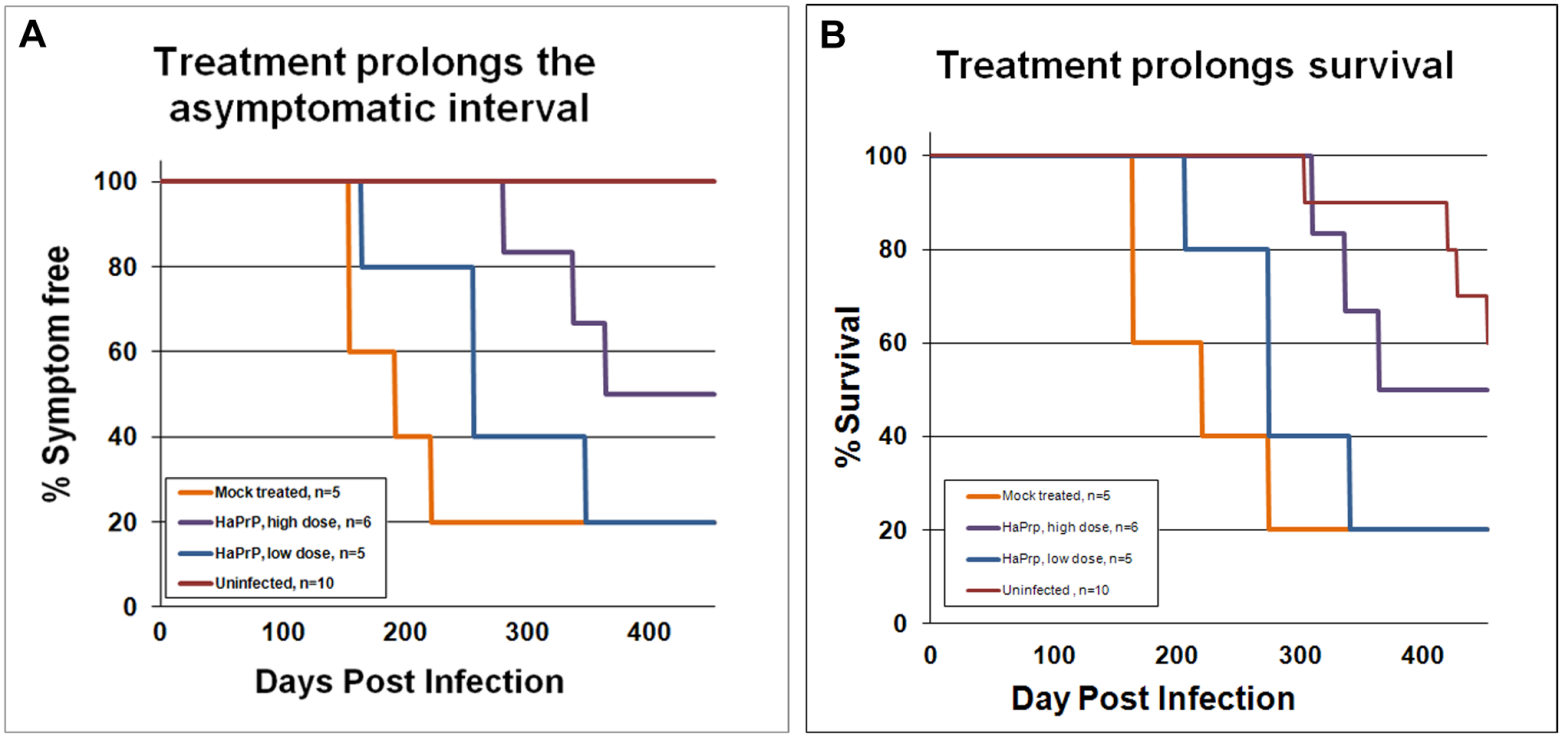
Treatment with heterologous recombinant HaPrP delayed the onset of symptoms and prolonged survival. Kaplan-Meier plots showing the time at which mock-treated (orange, n = 5), low-dose-treated (blue, n = 5), high-dose-treated mice (purple, n = 6) and uninfected (red, n = 10) developed A) detectable symptoms associated with scrapie infection, including ataxic gait, weight loss, and kyphosis, and B) time of survival. We tested for differences between groups using a modified version of the Gehan-Wilcoxon test and found a statistically significant difference between the mock infected group and the high dose group (*p* = 0.0348). The low-dose group was not significantly different than the mock-treated control group. The uninfected control mice showed significantly longer survival times than the three groups of infected mice; uninfected versus mock (p = 0.008), uninfected versus low dose (p = 0.006) and uninfected versus high dose (p = 0.0201).

In order to determine the efficacy of our treatment regime, we next examined whether treatment with HaPrP altered the time of presentation of first symptoms in the aged subset of mice infected with RML-Chandler scrapie. In mice the onset of scrapie symptoms is characterized by dull eyes, decreased motility, ataxic gait, hind limb paresis, kyphosis, weight loss, and flattened stature. After the onset of detectable prion disease associated symptoms in the study mice, symptoms typically became progressively worse over a course of three to four weeks and mice were euthanized. The HaPrP treated mice showed delayed onset of symptoms and prolonged survival relative to the mock-treated mice (Fig 4).

To test for differences in the survival distributions between groups we used the Peto-Peto modification of the Gehan-Wilcoxon test (a form of the log rank test that has the most power for detecting early differences). The uninfected control mice showed significantly longer survival times than the three groups of infected mice; uninfected versus mock (p = 0.008), uninfected versus low dose (p = 0.006) and uninfected versus high dose (p = 0.0201). This test also indicated no difference between the low dose and mock treated group (*p* = 0.219) and no difference between the low dose and high dose groups (*p* = 0.109). However, we did detect a significant difference between the high dose and mock treated group (*p* = 0.0348). When the study was terminated at 452 days post-infection, half of the high dose treated animals were still free of scrapie symptoms. These results support our hypothesis and indicate that recombinant hamster prion protein can effectively and significantly delay the onset of clinical symptoms and prolong survival in prion disease-infected mice.

As a side note, 2 of the 16 scrapie-infected mice that were aged, 1 from the high-dose-treated group and 1 from the mock-treated group, became obese and interestingly remained free of any apparent scrapie-associated symptoms at the end of the study.

To ascertain whether the obese mice mentioned above as well as other high-dose-treated mice that made it to the end of the study without detectable clinical symptoms had detectable PrP^res^ and were infected, we performed western blot analysis. The proteinase K treated brain homogenates from all four animals showed faint anti-PrP specific immunoreactive bands (S2 Fig), indicating that they were in fact infected.

## Discussion

### Treatment with heterologous prion protein did not prevent scrapie infection but it did slow disease progression and increase scrapie survival time

Western blot analysis demonstrated the presence of detectable PrP^res^ in all mice evaluated from all three treatment groups thus demonstrating active scrapie infection. This infection was associated with brain disease as evidenced by the presence of spongiosis and gliosis in all three treatment groups as determined by immuno-histological analyses. However, treatment with the heterologous hamster prion protein was effective in slowing disease progression and extending survival times. Although the numbers of animals in each group was small, similar trends were observed in both high and low dose treatment groups. These trends included increased incubation time and decreased pathology in both treatment groups. However, these changes were only statistically significant in the high dose treatment group. More meaningfully, survival times were increased in both treatment groups with a significant difference seen between the mock-treated and high-dose-treated mice.

Histopathological examination showed that the thalamus was relatively spared compared to other brain regions in treated mice compared to mock-treated mice. We do not know why the thalamus was spared, but speculate that possibly, 1) the HaPrP treatment preferentially inhibited PrP^sc^ formation in the thalamus, or 2) the spread of infection in the brain reached the thalamus last in these study animals.

### Heterologous PrP^C^ inhibits prion replication

The mechanism by which heterologous PrP^C^ inhibits PrP^res^ replication is unknown. It seems likely that the presence of heterologous PrP^C^ blocks the normal pathway for prion replication. A favored model for the PrP^C^ conversion process is a seeded polymerization model [48]. The rate limiting step of this model is the nucleation phase in which monomers undergo conformational change and self-associate to form oligomeric nuclei. In the second step of this process, these nuclei rapidly grow through the further addition of monomers, forming larger fibrils with subsequent fragmentation to build new nuclei [49]. The sequence of PrP^C^ may regulate either or both of these phases. *In vitro* studies suggest that heterologous proteins can bind to the oligomeric form and thereby interfere with conversion process [27]. These studies have also shown that this interference can occur with molar ratio of heterozygous to homozygous PrP^C^ as low as 1:1 consistent with the observation that heterozygosity for prion alleles can greatly influence disease susceptibility. For this reason we used the lowest dose of inoculum predicted to induce infection in 100% of the mice in order to maximize the chances that the recombinant hamster proteins could effectively inhibit PrP^res^ propagation. Our results did indeed show a dose dependent response with the higher treatment dose having a larger effect in delaying disease progression, indicating that stoichiometry may be important for this effect.

### Slower disease progression and increased survival were not the result of an immune response to foreign hamster prion protein

Another possible mechanism for the protective effect of heterologous prion protein is that it elicited an immune response that impeded PrP^res^ production. In order to test this hypothesis we utilized the Western blot technique to screen the plasma of treated and untreated mice for the presence of anti-hamster prion protein antibodies. Results from these studies failed to show antibody production in treated animals, that was not seen in negative control animals. No signal was detected in experimental or negative control animals with 1:500 or 1:1000 serum dilutions. Although some hamster reactive material was detected at low serum dilutions (1:200), this material was also seen in samples from negative control mock-infected as well as uninfectedmice indicating this reactivity was not due to the HaPrP treatment (data not shown).

### Heterologous prion protein may serve as a novel treatment for prion disease

The exact mechanism of action of this current treatment requires further investigation. One possibility is that the presence of heterologous PrP^C^ in the inoculum served to inactive the scrapie prion by binding to PrP^res^ and forming an inactive complex due to sequence incongruence. This suggests a potentially novel way to inactivate prions since disinfection of prions is extremely difficult. Another possibility is that injection of HaPrP into the mouse brain inhibited the *in vivo* conversion process. This raises the interesting prospect of treating prion diseases with heterologous prion protein. The treatment regime used in this study, intracerebral injection of heterologous PrP^C^ at the time of infection followed by oral ingestion of heterologous PrP^C^ is not ideal for treating patients with prion disease. However, since the presence of heterologous PrP^C^ slowed disease progression, more practical methods for treatment with heterologous PrP^C^ can be developed. Interestingly, chronic injection of a recombinant lentiviral vector expressing a dominant negative prion protein directly into the brains of prion disease infected mice at 80 and 90 days post-infection was shown to reduce astrocytic gliosis and extend the survival of these mice [50]. These results confirm our observations that heterologous PrP^C^ may be a useful therapy for prion diseases.

In summary, this study explored the innovative idea that heterologous normal cellular prion proteins (PrP^C^) from diverse species can be used to treat animals and humans infected with prion diseases. The results of this study suggest that treatment of scrapie-infected mice with bacterially expressed hamster prion proteins (HaPrP) was safe and effectively inhibited prion disease. Treated animals showed significantly delayed onset of motor deficits, significantly less gliosis, decreased spongiosis, decreased accumulation of protease resistant disease associated prion protein (PrP^res^), significant delays in onset of clinical symptoms, and significant increases in survival times relative to mock-treated mice. Mice tolerated treatment with hamster PrP^C^ without any apparent negative effects. These results provide proof of principle that recombinant hamster prion proteins can safely and effectively inhibit prion disease in mice, and suggest that hamster or other non-human prion proteins may be a viable treatment for prion diseases in humans. Furthermore, the concept behind this treatment might be widely applicable to other neurodegenerative diseases that involve protein misfolding, such as Alzheimer’s disease and Huntington’s chorea.

## Supporting Information

S1 Fig Histopathological assessment of seven brain regions in treated and mock-treated mice. A) spongiosis lesion profile. Brain region: 1 = Cerebral cortex dorsal to the septal nucleus, 2 = Cerebral cortex dorsal to the corpus callosum, 3 = Hippocampus, 4 = Thalamus, 5 = Hypothalamus, 6 = Cerebellar cortex, 7 = Medulla. B) Levels of GFAP+ cells per unit area, and C) Levels of iba1+ cells per unit area. For B) and C) Brain regions: 1 = Cerebral Cortex, 2 = Striatum, 3 = Thalamus, 4 = Hypothalamus, 5 = Hippocampus, 6 = Cerebellum, 7 = Medulla Error bars = Standard Error of the Mean. For each graph, the mock-treated group is blue, the low-dose-treated group is red, and the high-dose-treated group is green. Below each graph are representative images from mock-treated animals on the left and high-dose-treated animals on the right.
(TIF)

S2 Fig Mice that were symptom free at the end of the experiment demonstrated low but detectable PrP^res^. Western blot of PrP^res^ from archived frozen brain samples from scrapie infected mice that were symptom free at the end of the experiment. Mouse antibodies directed against PrP (SAF83) were used to detect PrP^C^ and Proteinase K treated (PK) PrP^res^. Left to right, gel lanes are as follows: 1. scrapie-infected mock-treated animal, 2–4. scrapie-infected high-dose-HaPrP treated animals, 5. Magic Mark XP molecular weight markers, 6. Scrapie-infected positive control inoculated with 1% RML Chandler (no PK), 7. scrapie-infected positive control (with PK), 8. Negative control animal mock-infected with 1% normal mouse brain (no PK), 9. Negative control (with PK). The ~31 kDa band observed in lanes with PK treated (+) samples is nonspecific based on comparison with the negative control in lane 9. The <30 kDa bands in lanes 1–4 are specific based on comparison with the positive control in lane 7 and absence of these bands in lane 9. Photoshop was used to remove two lanes containing irrelevant samples on the blot (indicated by white space).
(TIF)

S1 Checklist NC3Rs ARRIVE Guidelines Checklist. (PDF)
