# Supplementary figures and images for: Treatment of Prion Disease with Heterologous Prion Proteins
Source: PLoS One. 2015 Jul 2;10(7):e0131993. doi: 10.1371/journal.pone.0131993 (PMC4489745; doi:10.1371/journal.pone.0131993)

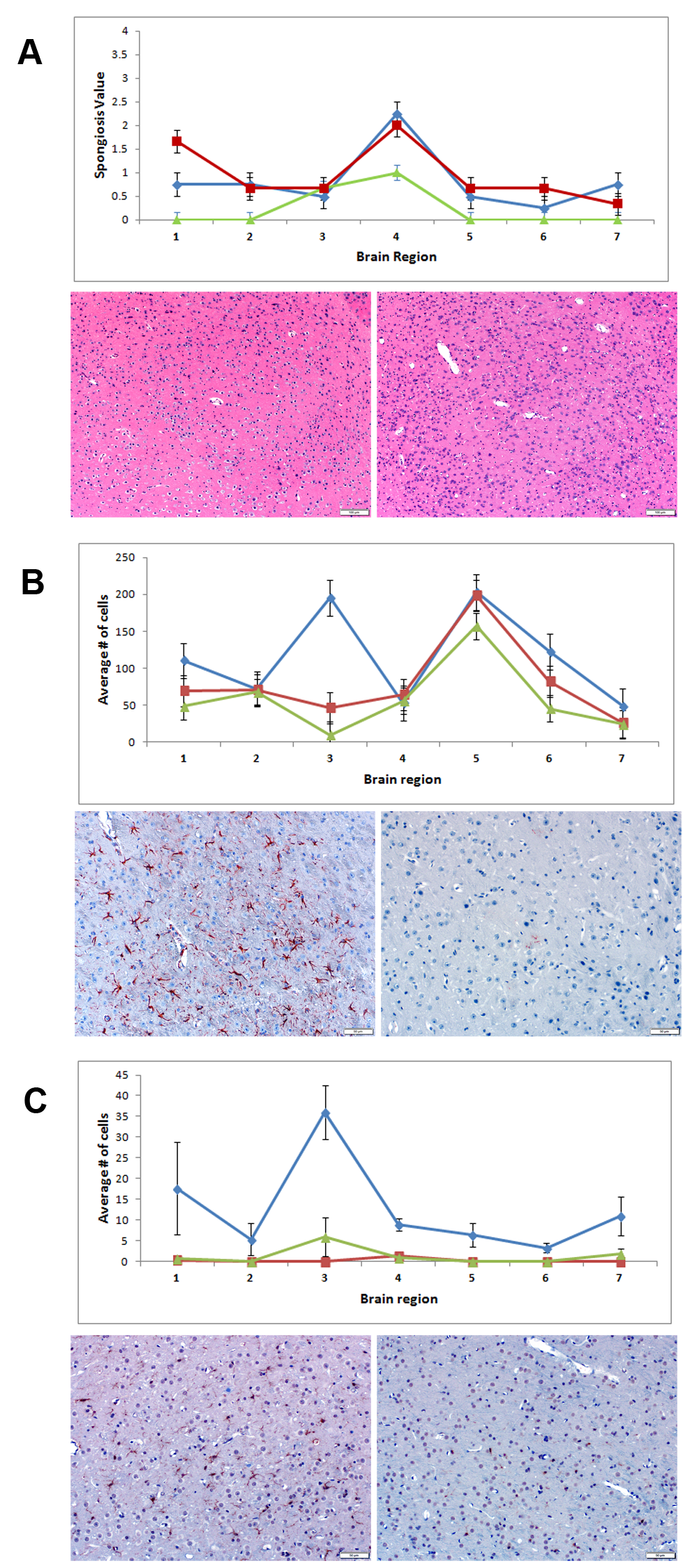

Supplement: S1 Fig — A) spongiosis lesion profile. Brain region: 1 = Cerebral cortex dorsal to the septal nucleus, 2 = Cerebral cortex dorsal to the corpus callosum, 3 = Hippocampus, 4 = Thalamus, 5 = Hypothalamus, 6 = Cerebellar cortex, 7 = Medulla. B) Levels of GFAP+ cells per unit area, and C) Levels of iba1+ cells per unit area. For B) and C) Brain regions: 1 = Cerebral Cortex, 2 = Striatum, 3 = Thalamus, 4 = Hypothalamus, 5 = Hippocampus, 6 = Cerebellum, 7 = Medulla Error bars = Standard Error of the Mean. For each graph, the mock-treated group is blue, the low-dose-treated group is red, and the high-dose-treated group is green. Below each graph are representative images from mock-treated animals on the left and high-dose-treated animals on the right. (TIF) [file pone.0131993.s001.tif]

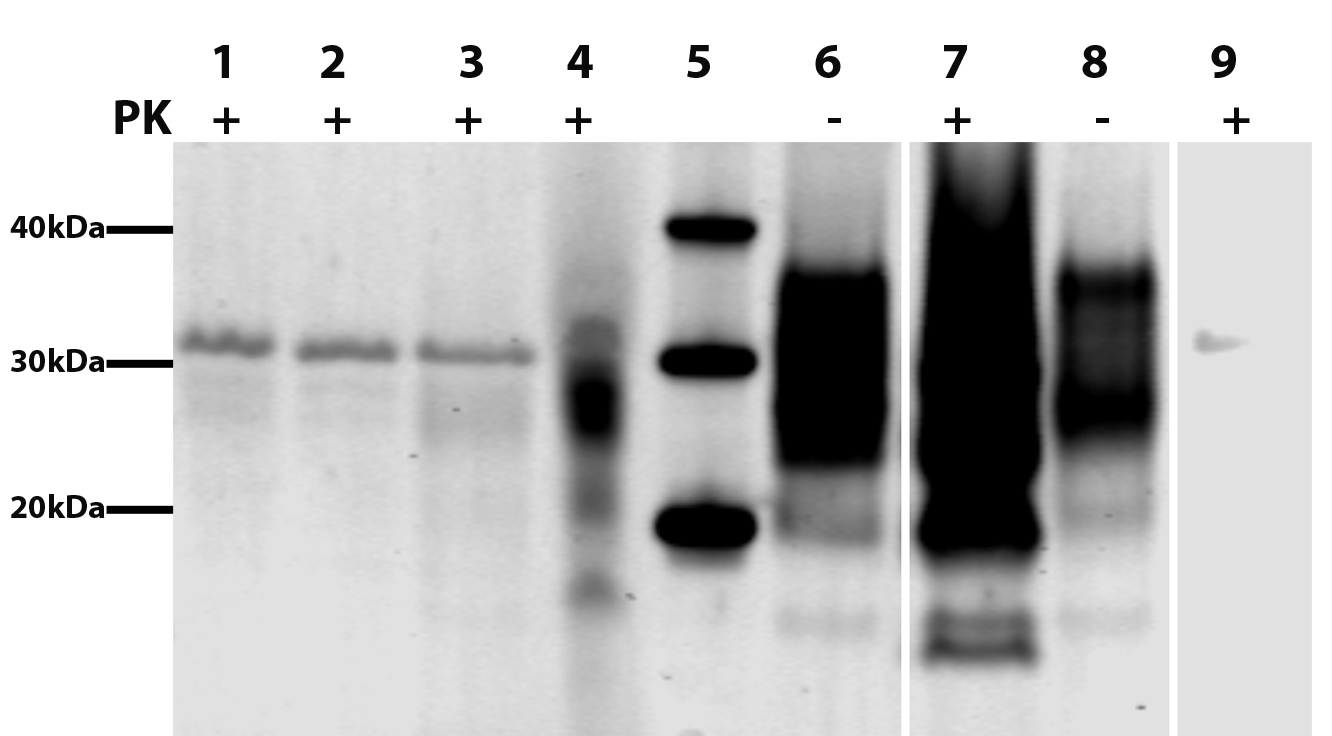

Supplement: S2 Fig — Western blot of PrPres from archived frozen brain samples from scrapie infected mice that were symptom free at the end of the experiment. Mouse antibodies directed against PrP (SAF83) were used to detect PrPC and Proteinase K treated (PK) PrPres. Left to right, gel lanes are as follows: 1. scrapie-infected mock-treated animal, 2–4. scrapie-infected high-dose-HaPrP treated animals, 5. Magic Mark XP molecular weight markers, 6. Scrapie-infected positive control inoculated with 1% RML Chandler (no PK), 7. scrapie-infected positive control (with PK), 8. Negative control animal mock-infected with 1% normal mouse brain (no PK), 9. Negative control (with PK). The ~31 kDa band observed in lanes with PK treated (+) samples is nonspecific based on comparison with the negative control in lane 9. The <30 kDa bands in lanes 1–4 are specific based on comparison with the positive control in lane 7 and absence of these bands in lane 9. Photoshop was used to remove two lanes containing irrelevant samples on the blot (indicated by white space). (TIF) [file pone.0131993.s002.tif]
