# Supplementary material for: Treatment of Prion Disease with Heterologous Prion Proteins
Source: PLoS One. 2015 Jul 2;10(7):e0131993. doi: 10.1371/journal.pone.0131993 (PMC4489745; doi:10.1371/journal.pone.0131993)
Supplement: S1 Checklist — (PDF) [file pone.0131993.s003.pdf]

The ARRIVE Guidelines Checklist

Animal Research: Reporting In Vivo Experiments

Carol Kilkenny[1], William J Browne[2], Innes C Cuthill[3], Michael Emerson[4] and Douglas G Altman[5]

[1]The National Centre for the Replacement, Refinement and Reduction of Animals in Research, London, UK, [2]School of Veterinary Science, University of Bristol, Bristol, UK, [3]School of Biological Sciences, University of Bristol, Bristol, UK, [4]National Heart and Lung Institute, Imperial College London, UK, [5]Centre for Statistics in Medicine, University of Oxford, Oxford, UK.

	ITEM	RECOMMENDATION	Section/ Paragraph
Title	1	Provide as accurate and concise a description of the content of the article as possible.	
Abstract	2	Provide an accurate summary of the background, research objectives, including details of the species or strain of animal used, key methods, principal findings and conclusions of the study.	
**INTRODUCTION**			
Background	3	a. Include sufficient scientific background (including relevant references to previous work) to understand the motivation and context for the study, and explain the experimental approach and rationale.<br>b. Explain how and why the animal species and model being used can address the scientific objectives and, where appropriate, the study's relevance to human biology.	
Objectives	4	Clearly describe the primary and any secondary objectives of the study, or specific hypotheses being tested.	
**METHODS**			
Ethical statement	5	Indicate the nature of the ethical review permissions, relevant licences (e.g. Animal [Scientific Procedures] Act 1986), and national or institutional guidelines for the care and use of animals, that cover the research.	
Study design	6	For each experiment, give brief details of the study design including:<br>a. The number of experimental and control groups.<br>b. Any steps taken to minimise the effects of subjective bias when allocating animals to treatment (e.g. randomisation procedure) and when assessing results (e.g. if done, describe who was blinded and when).<br>c. The experimental unit (e.g. a single animal, group or cage of animals).<br>A time-line diagram or flow chart can be useful to illustrate how complex study designs were carried out.	
Experimental procedures	7	For each experiment and each experimental group, including controls, provide precise details of all procedures carried out. For example:<br>a. How (e.g. drug formulation and dose, site and route of administration, anaesthesia and analgesia used [including monitoring], surgical procedure, method of euthanasia). Provide details of any specialist equipment used, including supplier(s).<br>b. When (e.g. time of day).<br>c. Where (e.g. home cage, laboratory, water maze).<br>d. Why (e.g. rationale for choice of specific anaesthetic, route of administration, drug dose used).	
Experimental animals	8	a. Provide details of the animals used, including species, strain, sex, developmental stage (e.g. mean or median age plus age range) and weight (e.g. mean or median weight plus weight range).<br>b. Provide further relevant information such as the source of animals, international strain nomenclature, genetic modification status (e.g. knock-out or transgenic), genotype, health/immune status, drug or test naïve, previous procedures, etc.	

Housing and husbandry	9	Provide details of:<br>a. Housing (type of facility e.g. specific pathogen free [SPF]; type of cage or housing; bedding material; number of cage companions; tank shape and material etc. for fish).<br>b. Husbandry conditions (e.g. breeding programme, light/dark cycle, temperature, quality of water etc for fish, type of food, access to food and water, environmental enrichment).<br>c. Welfare-related assessments and interventions that were carried out prior to, during, or after the experiment.	
Sample size	10	a. Specify the total number of animals used in each experiment, and the number of animals in each experimental group.<br>b. Explain how the number of animals was arrived at. Provide details of any sample size calculation used.<br>c. Indicate the number of independent replications of each experiment, if relevant.	
Allocating animals to experimental groups	11	a. Give full details of how animals were allocated to experimental groups, including randomisation or matching if done.<br>b. Describe the order in which the animals in the different experimental groups were treated and assessed.	
Experimental outcomes	12	Clearly define the primary and secondary experimental outcomes assessed (e.g. cell death, molecular markers, behavioural changes).	
Statistical methods	13	a. Provide details of the statistical methods used for each analysis.<br>b. Specify the unit of analysis for each dataset (e.g. single animal, group of animals, single neuron).<br>c. Describe any methods used to assess whether the data met the assumptions of the statistical approach.	
RESULTS			
Baseline data	14	For each experimental group, report relevant characteristics and health status of animals (e.g. weight, microbiological status, and drug or test naïve) prior to treatment or testing. (This information can often be tabulated).	
Numbers analysed	15	a. Report the number of animals in each group included in each analysis. Report absolute numbers (e.g. 10/20, not 50%[2]).<br>b. If any animals or data were not included in the analysis, explain why.	
Outcomes and estimation	16	Report the results for each analysis carried out, with a measure of precision (e.g. standard error or confidence interval).	
Adverse events	17	a. Give details of all important adverse events in each experimental group.<br>b. Describe any modifications to the experimental protocols made to reduce adverse events.	
DISCUSSION			
Interpretation/ scientific implications	18	a. Interpret the results, taking into account the study objectives and hypotheses, current theory and other relevant studies in the literature.<br>b. Comment on the study limitations including any potential sources of bias, any limitations of the animal model, and the imprecision associated with the results[2].<br>c. Describe any implications of your experimental methods or findings for the replacement, refinement or reduction (the 3Rs) of the use of animals in research.	
Generalisability/ translation	19	Comment on whether, and how, the findings of this study are likely to translate to other species or systems, including any relevance to human biology.	
Funding	20	List all funding sources (including grant number) and the role of the funder(s) in the study.	

References:
1. Kilkenny C, Browne WJ, Cuthill IC, Emerson M, Altman DG (2010) Improving Bioscience Research Reporting: The ARRIVE Guidelines for Reporting Animal Research. *PLoS Biol* 8(6): e1000412. doi:10.1371/journal.pbio.1000412
2. Schulz KF, Altman DG, Moher D, the CONSORT Group (2010) CONSORT 2010 Statement: updated guidelines for reporting parallel group randomised trials. *BMJ* 340:c332.

1. Treatment of Prion Disease with Heterologous Prion Proteins
2. Prion diseases such as Creutzfeldt-Jakob disease in humans, bovine spongiform encephalopathy in cattle, and scrapie in sheep are fatal neurodegenerative diseases for which there is no effective treatment. The pathology of these diseases involves the conversion of a protease sensitive form of the cellular prion protein (PrPC) into a protease resistant infectious form (PrP^{sc} or PrPres). Both *in vitro* (cell culture and cell free conversion assays) and *in vivo* (animal) studies have demonstrated the strong dependence of this conversion process on protein sequence homology between the initial prion inoculum and the host's own cellular prion protein. The presence of non-homologous (heterologous) proteins is often inhibitory to this conversion process. We hypothesize that the presence of heterologous prion proteins from one species might therefore constitute an effective treatment for prion disease in another species. To test this hypothesis, we infected anesthetized C57BL/6 female mice intracerebrally with murine adapted RML-Chandler scrapie and treated them with heterologous prion protein (purified bacterially expressed recombinant hamster prion protein) or vehicle alone. Treated animals demonstrated reduced astrocytosis, decreased accumulation of protease-resistant disease-associated prion protein with delayed onset of clinical symptoms and motor deficits. This was concomitant with significantly increased survival times relative to mock treated animals. These results provide proof of principle that recombinant hamster prion proteins can effectively and safely inhibit prion disease in mice, and suggest that hamster or other non-human prion proteins may be a viable treatment for prion diseases in humans.
3. Prion diseases, also known as transmissible spongiform encephalopathies (TSE), are rare progressive neurodegenerative diseases that are transmissible between species[1-3]. These diseases include Creutzfeldt-Jakob disease (CJD) in humans; bovine spongiform encephalopathy (BSE) in cattle[4]; chronic wasting disease (CWD) in deer and elk[5]; and scrapie in sheep, goats, and experimentally infected rodents[1]. Prion diseases belong to a growing family of disorders that are attributed to misfolding and aggregation of proteins, including Alzheimer's, Parkinson's disease and systemic amyloidosis[6,7]Some distinguishing features of prion disease are their wide phenotypic variety and their multiple methods of acquisition (sporadic, genetic or acquired) [1,8,9]. The infectious agent in these diseases are prions (proteinaceous infectious particles) [2]. Prion diseases are believed to involve misfolding of an endogenous cellular prion protein, PrPC, into a variant self-replicating isoform, PrPres[10]. The mechanism of this is uncertain, but it is believed that an aggregate of PrPres protein binds the cellular PrPC and catalyzes its conversion to an infectious form[11]. The misfolding and accumulation of prion proteins is thought to be the basis of prion disease pathogenesis and infectivity[2]. The PrPC gene encodes a 253 amino acid long protein, which is post-translationally processed to an approximately 210 amino acid long protein via cleavage at both its N and C terminus[1,3,8,12]. Structural studies suggest that it is arranged with a disordered amino-terminal tail and a globular C-terminal domain composed of three □-helices and a short anti-parallel □□sheet[13,14]. It is anchored to the outer cell surface membrane via a glycosylphosphatidylinositol (GPI) anchor which helps tether the protein to the outer cell surface membrane[15]. Whereas PrPC exists predominately as a monomer/dimer in an alpha helical configuration, the variant PrPres is aggregated in nature and exists predominately in a □-pleated sheet rich conformation[16,17]. This aggregated misfolded PrPres state is characterized by resistance to protease degradation and chemical disinfection[18]. It is proposed that the normal replication of PrPres is dependent on recruitment of PrPC into this altered PrPres configuration. The primary structure of host PrPC is a major determinant of prion disease susceptibility. Transgenic mice that lack PrPC are resistance to prion infection[19]. A high degree of sequence identity between the infecting prion and the host PrPC is often necessary for efficient prion replication[8,20,21]. Moreover, differences in the PrPC sequence have been proposed to be involved in resistance to cross species infection (species barriers) and prion strains[22,23]. However, this effect is not strictly dependent on amino acid homology and appears to be more dependent on subtle structural variations, most notably differences within the loop/turn structures[16]. Experiments in transgenic mice, tissue culture cells and cell-free systems have identified the middle third region of the prion protein as being important for the autocatalytic conversion process[22]. Polymorphisms in this region of the PrP gene can confer resistance to prion disease, whereas homology at critical amino acid residues has been demonstrated to facilitate cross-species transmission of prion disease. Transgenic mice expressing a variant mouse PrP containing three amino acid substitutions in the □2-□2 loop completely resisted infection

with two different strains of prions[24]. There is currently no effective treatment for prion diseases in humans, and these diseases in humans are always fatal. Drugs have been identified which show some efficacy in treating prion diseases in tissue culture systems or in whole animal systems[25,26]. Two of these compounds, quinacrine and pentosan polysulfate have been used as compassionate therapy of patients with CJD or vCJD, however, no therapeutic benefit was seen [27,28]. Other treatment strategies for prion diseases have been attempted including vaccination and immunotherapy, but these strategies have had limited success.[29,30] Another recent therapeutic target for prion disease is the unfolded protein response[31] since misfolding of prion proteins during disease development stimulates the activation of the unfolding response pathway. Chemical inhibition of this pathway has been demonstrated to be neuroprotective and to abrogate disease development in prion-infected mice[32]. Another recent treatment strategy has employed lentivirus vectors expressing silencing RNAs directed against the cellular form of the prion protein[33]. These lentivirus vectors were used to transduce mouse embryonic stem cells and create chimeric mice expressing various levels of the silencing RNAs. Scrapie survival times were extended in those mice that were highly chimeric for the transgene and that showed reduced PrPC expression in the brain. We hypothesize that heterologous prion proteins can be used as a viable treatment for prion diseases. In other words, normal prion proteins from one species can be used to treat prion disease in another species. The rationale for this hypothesis comes from several previously published studies. Induced expression of hamster prions in scrapie-infected mouse cells almost completely eliminates the accumulation of the misfolded prion protein isoform (PrPres) in contrast to cell lines not expressing the hamster gene[34]. This suggests that heterologous hamster prion proteins may actually inhibit PrPres production in the scrapie-infected cells. Similarly, scrapie-infected mouse cells that are induced to express a rabbit prion gene produce substantially less PrPres as compared to mouse cells that do not express rabbit prion proteins[35], suggesting that rabbit prion proteins may interfere with mouse PrPres formation. Inhibition of PrPres formation by heterologous prion proteins has also been observed in studies of transgenic mice. In transgenic mice expressing both endogenous mouse prion proteins as well as an exogenous hamster prion gene, onset of disease and death after mouse scrapie infection is significantly delayed compared to wild-type mice[36]. Furthermore, transgenic mice expressing human PrPC are resistant to infection with human prions[37], but become susceptible upon ablation of the mouse PrPC gene[38] indicating that the mouse prion proteins inhibit the propagation of human prions. The goal of this study was to test the proof of principle that heterologous prion proteins can be used to effectively treat prion disease.Scrapie infection in mice has been well studied [39]and intracerebral inoculation of mice with low doses of scrapie inoculum offers a model of iatrogenic prion disease transmission that occurs in humans. The mouse scrapie model system in conjunction with recombinant hamster prion proteins offers a relatively safe and effective system in which to test the hypothesis that heterologous prion proteins can be used to effectively treat prion diseases. For this study we evaluated the effectiveness of treating scrapie-infected mice with bacterially expressed and purified recombinant hamster prion proteins. The results of this study provide proof of principal that heterologous prion proteins can be used safely and effectively to treat prion diseases.

4. The primary objectives of this study were to determine whether treatment with HaPrP prolonged the lives of scrapie-infected mice. The secondary objectives of this study were to dermine whether treatment with HaPrP delayed the onset of PrPres formation, astrocytosis, motor functional loss, and disease associated symptoms.
5. The University of Minnesota has an approved Animal Welfare Assurance #A3456-01 on file with the NIH Office of Laboratory Animal Welfare and complies with the USDA Animal Welfare Act Regulations, and the Public Health Service Policy on Humane Care and Use of Laboratory Animals. All animal studies at both the University of Minnesota and the Rocky Mountain Laboratories were carried out in strict accordance with the recommendations in the Guide for the Care and Use of Laboratory Animals of the National Institutes of Health. The Academic Health Center and the Rocky Mountain Laboratories are fully accredited by the Association for the Assessment and Accreditation of Laboratory Animal Care, International. The University of Minnesota Institutional Animals Care and Use Committee (IACUC) approved animal protocol (#0702A03021) and the Rocky Mountain Laboratories Animal Care and Use Committee approved Protocol 03-06 for use in the study.
6. Four week-old C57BL/6 female mice were separated to three treatment groups with 13 animals each. Recombinant hamster prion proteins (HaPrP) or vehicle alone were

administered both at the time of inoculation with of scrapie and orally the following day.

Table 1. Distribution of mice in study

Group	Total # mice	Died after injection	Pre-clinical 108 dpi		Clinical symptoms
			Histology	Prp^{sc} detection	Motor skills Symptoms Survival
Mock-treated	13	1	3	4	5
Low dose treated	13	1	3	4	5
High dose treated	13	0	3	4	6
Not infected or treated	10				10

7. **Treatment of scrapie-infected mice.** Four week-old C57BL/6 female mice were separated to three treatment groups with 13 animals each. Recombinant hamster prion proteins (HaPrP) or vehicle alone were administered both at the time of inoculation with of scrapie and orally the following day. Mice were anaesthetized with isoflurane (UMN Research Animal Resource Veterinarian recommended method) and intracerebrally inoculated with 5 µl of a 0.01% RML-Chandler strain scrapie brain homogenate (obtained from mice with symptomatic scrapie infection) diluted in PBS containing 2% fetal calf serum, plus 45ul of either HaPrP (0.7mg/ml) for high dose treatment (this was the highest concentration we achieved in prepping HaPrP and why we used this concentration), HaPrP (0.45 mg/ml) for low dose treatment, or 45 ul of vehicle only (10 mM sodium acetate, pH 5) for mock-treated mice. The titer of the scrapie inoculum used is estimated to be at least as high as the stock of RML-Chandler used to derive this inoculum, which was $2X10^8$ infectious units/gram of brain[41]. The following day, mice were treated orally with 100ul of recombinant proteins or vehicle alone for mock-treated mice. Animals were evaluated weekly during the first months and then daily in later months by a veterinarian for signs of scrapie-related symptoms including ataxic gait, hind limb paresis, decreased motility, dull eyes, flattened stature, weight loss, and kyphosis. At the same time, animals were also evaluated for signs of non-scrapie related disease or discomfort. Animals with symptoms of scrapie, or otherwise found to be suffering, as determined by the veterinarian, were euthanized with $C0_2$. Three of the 16 aged study mice were euthanized due to the development of severe dermatitis. The development of severe dermatitis is common in C57BL/6 mice[44] and not thought to be attributed to the scrapie infection or our HaPrP treatment. Animal infections were done in a biosafety cabinet between 8 AM and 2PM. **Hanging wire assay** An adaptation of the hanging wire assay[42] was used as a simple and atraumatic assessment of neuromuscular function during the latter portion of the study. Mice used their forelimbs or hindlimbs in any combination to grasp a wire mesh suspended 20 cm above a cushioned cage bottom. A series of three 120-second trials was conducted on each test day and the times until mice dropped were averaged. Trials were done between 8 AM and 2PM.
8. Four week-old C57BL/6 female mice were separated to three treatment groups with 13 animals each. An additional 10 retired breeder female C57BL/6 mice were untreated and uninfected and used as a control. Animal weight was not determined, nor used as an inclusion or exclusion criteria. Mice were purchased from Harlan Laboratories, were healthy, not gene modified, and drug naive.
9. Mice were housed in the University of Minnesota Research Animal Resources facilities in the Veterinary Science building in a USDA-approved BSL-2 mouse room, and were cared for by RAR staff and veterinarians, as well as by Dr. Hyeon Kim DVM (Skinner lab scientist). All animals at the University of Minnesota are maintained and cared for as outlined in the Guide for the Care and Use of Laboratory Animals (National Institutes of Health Publication 78-23). The mouse room was specially designated for animal care and maintenance of scrapie-infected mice. Each room in this areas is well ventilated, air-conditioned and provided with an independently adjustable, light-dark cycle system and temperature regulation system. The rooms and animal cages were cleaned daily, and the animals were provided with fresh food and water on a daily basis. Cages contained wood shavings and bedding material, and 4-5 mice per cage. The University of Minnesota is a registered Research Facility under the Animal Welfare Act. It has a current Letter of Assurance on file with the Office for Protection from Research Risks, in compliance with NIH Policy. The

Animal Care facilities are under the direction of a Doctor of Veterinary Medicine and are staffed by veterinarians with training and experience in laboratory animal medicine and surgery, clinical care and diagnostic pathology. UMN Research Animal Resources veterinarians inspected animals weekly. In addition, our staff veterinarian, Dr. Hyeon Kim assessed the health and well being of the mice daily during the first week post-infection, then weekly, and then again daily after 200 days post-infection to determine when the onset of clinical symptoms began. At the same time, animals were also evaluated for signs of non-scrapie related disease or discomfort. Animals with symptoms of scrapie, or otherwise found to be suffering, as determined by the overseeing veterinarian, were euthanized with CO_2. Mice were anaesthetized with isoflurane prior to intracerebral inoculation with scrapie. Animals with symptoms of scrapie, or otherwise found to be suffering, as determined by the veterinarian, were euthanized with CO_2. At experimental endpoints mice were similarly euthanized with CO_2.

10. Four week-old C57BL/6 female mice were separated to three treatment groups with 13 animals each. Recombinant hamster prion proteins (HaPrP) or vehicle alone were administered both at the time of inoculation with of scrapie and orally the following day. Mice were anaesthetized with isoflurane and intracerebrally inoculated with 5 µl of a 0.01% RML-Chandler strain scrapie brain homogenate (obtained from mice with symptomatic scrapie infection) diluted in PBS containing 2% fetal calf serum, plus 45ul of either HaPrP (0.7mg/ml) for high dose treatment, HaPrP (0.45 mg/ml) for low dose treatment, or 45 ul of vehicle only (10 mM sodium acetate, pH 5) for mock-treated mice. The titer of the scrapie inoculum used is estimated to be at least as high as the stock of RML-Chandler used to derive this inoculum, which was $2X10^8$ infectious units/gram of brain[41]. The following day, mice were treated orally with 100ul of recombinant proteins or vehicle alone for mock-treated mice. An additional group of 10 untreated and uninfected mice was included as a control. This was a pilot study without preliminary data needed to statistically determine animal numbers.
11. Mice were randomly allocated into experimental groups.
12. The primary objectives of this study were to determine whether treatment with HaPrP prolonged the lives of scrapie-infected mice. The secondary objectives of this study were to determine whether treatment with HaPrP delayed the onset of PrPres formation, astrocytosis, motor functional loss, and disease associated symptoms.
13. To test for differences in survival time distributions between animal groups the Peto Peto modification of the Gehan-Wilcoxon test was used [43](this test is one version of the log rank test). This test is suited to detecting early differences in the survival distribution in the presence of right censored data. There is right censoring in this data set as not all animals had been diagnosed with symptoms of scrapie at termination of the study. To test for differences in the temporal trajectories of performance on the wire hanging assay, a mixed model was used with indicator variables for group membership, a linear time trend, interactions between the group indicator variables and the time trend and animal specific random effects. The inclusion of random effects allows us to correctly model the dependence among observations from the same animal at different time points. These models were fit using restricted maximum likelihood and the Wald test associated with the slopes for the interaction between time and group membership. This provides a test for differences in the rate of decline in performance on this assay. One of the most important aspects of using mixed models in the context of longitudinal data analysis is that all of the data is used, and, in particular, specific time points are not selected by the data analyst (as this obviously biases the analysis). Since the data are necessarily values between 0 and 120, we first divide all of the data by 120 then use a logistic transformation to these observations to improve their approximation to normality. The Wilcoxon rank sum was used to test for differences between 2 groups except for the astrocytosis analysis where the sample size was too small for this test to have any power to detect a significant difference. For that analysis, 2 sample *t*-tests were used on the logarithm of the levels to improve the approximation to normality.All statistical calculations were conducted using the software R version 2.10.1. The R packages survival and nlme had the functions necessary for this analysis.
14. All mice appeared healthy at the beginning of the experiment.
15. The numbers of animals for each group are indicated in the table below.

Table 1. Distribution of mice in study

Group	Total # mice		Pre-clinical 108 dpi		Clinical symptoms
		Died after injection	Histology	Prp^{sc} detection	Motor skills Symptoms Survival
Mock-treated	13	1	3	4	5
Low dose treated	13	1	3	4	5
High dose treated	13	0	3	4	6
Not infected or treated	10				10

16. **Figure 1. Reduced PrPres accumulation in brain and spleen of high-dose-treated mice.** Western blot analysis of prion protein from the brain (A) and spleen (B) from scrapie-infected mice that were mock-treated with vehicle alone, treated with a relatively low dose of hamster prion protein, or treated with a relatively high dose of hamster prion protein, collected at 108 days post-scrapie infection. Mouse antibodies directed against PrP (SAF83) were used to detect PrPC and Proteinase K treated (PK) PrPres. GAPDH staining was used as a loading control. The average density of PrPres bands for each group is shown for the brain (C) and spleen (D). Significantly lower levels of PrPres were observed in the high-dose treated animals relative to mock-treated animals (*p*=0.0286 by the Wilcoxon rank sum test, also, see Figure 3). There was no observed difference between the low dose and mock infected groups (*p*=0.686 by the Wilcoxon rank sum test). **Figure 2. Decreased astrocytosis in brains of high-dose treated mice.** Anti-GFAP antibody (brown) and hematoxylin (blue) staining in A) whole brain and B) enlargement showing thalamus. For this analysis, regions of brain not present in all sections were removed (including cerebellum, brainstem, and olfactory bulb). Levels of GFAP staining are shown for C) whole brain, D) hippocamus, and E) thalamus. The high-dose-treated mice showed a trend of less GFAP accumulation in the brain compared to mock-treated mice (*p*=0.103 by a 2 sample *t*-test on the logarithms of the levels), whereas no difference was detected between the low-dose-treated and mock infected mice (*p*=0.906 again by a 2 sample *t*-test). Decreases in high-dose treated verses mock-treated mice were most pronounced in the thalamus (*p*=0.000557 by a 2 sample *t*-test); no significant differences in staining were seen amongst groups in the hippocampus (*p*=0.825 for the high-dose group compared to the mock treated group and *p*=0.839 for the low-dose group compared to the mock treated group, again with a 2 sample *t*-test). **Figure 3. Treatment with heterologous recombinant HaPrP delayed loss of motor function.** The hanging wire assay was used as an objective measure of motor coordination and muscle strength. The graphs show the results of A) mock-treated mice, B) uninfected age matched mice, C) low-dose-treated mice, and D) high-dose-treated mice. The x-axis shows the day post-infection and the Y-axis shows the time of latency to fall from a wire grid in a 120 second trial. Individual averages of triplicate trials are shown with error bars showing the standard deviation. Mixed model analysis (which uses the data from all time points to test for differences between groups) finds significant differences between both treatment dose groups and the mock infected group (*p*<0.001 for both tests).

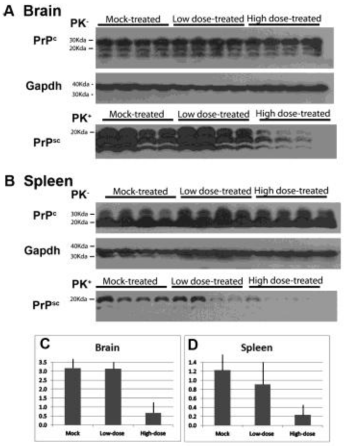

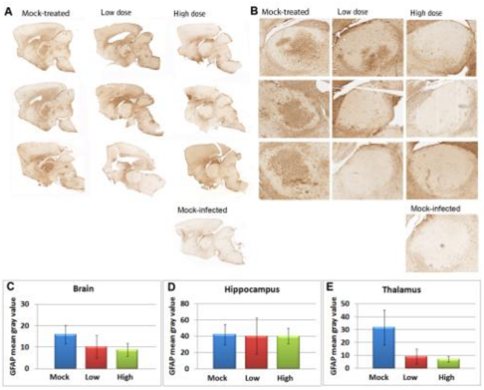

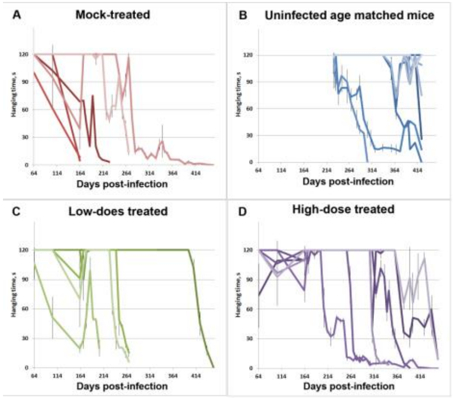

Significant differences (p<0.001) were also seen between the three groups (mock, low dose, high dose) and the uninfected age matched mice. **Figure 4. Treatment with heterologous recombinant HaPrP delayed the onset of symptoms and prolonged survival.** Kaplan-Meier plots showing the time at which mock-treated (orange, n=5), low-dose-treated (blue, n=5), high-dose-treated mice (purple, n=6) and uninfected (red, n=10) developed A) detectible symptoms associated with scrapie infection, including ataxic gait, weight loss, and kyphosis, and B) time of survival. We tested for differences between groups using a modified version of the Gehan-Wilcoxon test and found a statistically significant difference between the mock infected group and the high dose group (*p*=0.0348). The low-dose group was not significantly different than the mock-treated control group. The uninfected control mice showed significantly longer survival times than the three groups of infected mice; uninfected versus mock (p=0.008), uninfected versus low dose (p=0.006) and uninfected versus high dose (p=0.0201).

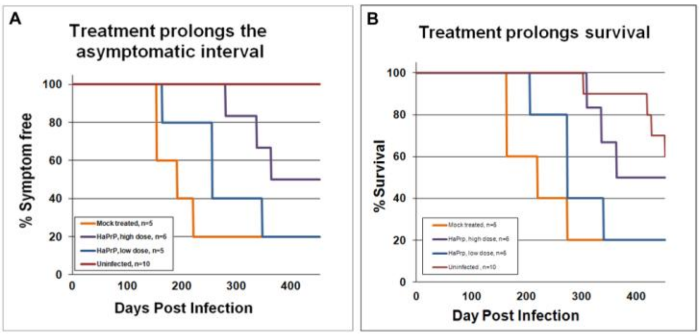

17. Three of the 16 aged study mice were euthanized due to the development of severe dermatitis. The development of severe dermatitis is common in C57BL/6 mice[44] and not thought to be attributed to the scrapie infection or our HaPrP treatment.
18. Treatment with heterologous prion protein did not prevent scrapie infection but it did slow disease progression and increase scrapie survival time. Western blot analysis demonstrated the presence of detectible PrPres in all mice evaluated from all three treatment groups thus demonstrating active scrapie infection. This infection was associated with brain disease as evidenced by the presence of astrocytosis in all three treatment groups as determined by immuno-histological analyses. However, treatment with the heterologous hamster prion protein was effective in slowing disease progression and extending survival times. Although the numbers of animals in each group was small, similar trends were observed in both high and low dose treatment groups. These trends included increased incubation time and decreased pathology in both treatment groups. However, these changes were only statistically significant in the high dose treatment group. More meaningfully, survival times were increased in both treatment groups with a significant difference seen between the mock treated and high dose treated mice. Group sizes in this study were relatively small, but produced significantly meaningful data. These data as a basis to statistically determine appropriate sample sizes to potentially reduce or minimize animals numbers for future studies.
19. Heterologous prion protein may serve as a novel treatment for prion disease. The exact mechanism of action of this current treatment requires further investigation. One possibility is that the presence of heterologous PrPC in the inoculum served to inactive the scrapie prion by binding to PrPres and forming an inactive complex due to sequence incongruency. This suggests a potentially novel way to inactivate prions since disinfection of prions is extremely difficult. Another possibility is that injection of HaPrP into the mouse brain inhibited the in vivo conversion process. This raises the interesting prospect of treating prion diseases with heterologous prion protein. The treatment regime used in this study, intracerebral injection of heterologous PrPC at the time of infection followed by oral ingestion of heterologous PrPC is not ideal for treating patients with prion disease. However, since the presence of heterologous PrPC slowed disease progression, more practical methods for treatment with heterologous PrPC can be developed. Interestingly, chronic injection of a recombinant lentiviral vector expressing a dominant negative prion protein directly into the brains of prion disease infected mice at 80 and 90 days post-infection was shown to reduce astrocytic gliosis and extend the survival of these mice[48]. These results confirm our observations that heterologous PrPC may be a useful therapy for prion diseases.
20. This project was funded by a small pilot grant from the University of Minnesota. The funders had no role in the study besides funding.

References

1. Caughey B, Chesebro B. Transmissible spongiform encephalopathies and prion protein interconversions. *Adv Virus Res*. 2001;56:277-311.

2. Prusiner SB. Novel proteinaceous infectious particles cause scrapie. *Science*. 1982;216(4542):136-144.

3. Aguzzi A, Polymenidou M. Mammalian prion biology: One century of evolving concepts. *Cell*. 2004;116(2):313-327.

4. Hope J, Reekie LJ, Hunter N, et al. Fibrils from brains of cows with new cattle disease contain scrapie-associated protein. *Nature*. 1988;336(6197):390-392. doi: 10.1038/336390a0 [doi].

5. Williams ES, Young S. Chronic wasting disease of captive mule deer: A spongiform encephalopathy. *J Wildl Dis*. 1980;16(1):89-98.

6. Soto C. Unfolding the role of protein misfolding in neurodegenerative diseases. *Nat Rev Neurosci*. 2003;4(1):49-60. doi: 10.1038/nrn1007 [doi].

7. Hetz C, Soto C. Protein misfolding and disease: The case of prion disorders. *Cell Mol Life Sci*. 2003;60(1):133-143.

8. Prusiner SB. Prions. *Proc Natl Acad Sci U S A*. 1998;95(23):13363-13383.

9. Poggiolini I, Saverioni D, Parchi P. Prion protein misfolding, strains, and neurotoxicity: An update from studies on mammalian prions. *Int J Cell Biol*. 2013;2013:910314. doi: 10.1155/2013/910314 [doi].

10. Moore RA, Taubner LM, Priola SA. Prion protein misfolding and disease. *Curr Opin Struct Biol*. 2009;19(1):14-22. doi: 10.1016/j.sbi.2008.12.007 [doi].

11. Caughey B. Prion protein conversions: Insight into mechanisms, TSE transmission barriers and strains. *Br Med Bull*. 2003;66:109-120.

12. Cobb NJ, Surewicz WK. Prion diseases and their biochemical mechanisms. *Biochemistry*. 2009;48(12):2574-2585. doi: 10.1021/bi900108v [doi].

13. Riek R, Hornemann S, Wider G, Glockshuber R, Wuthrich K. NMR characterization of the full-length recombinant murine prion protein, mPrP(23-231). *FEBS Lett*. 1997;413(2):282-288. doi: S0014-5793(97)00920-4 [pii].

14. Riek R, Hornemann S, Wider G, Billeter M, Glockshuber R, Wuthrich K. NMR structure of the mouse prion protein domain PrP(121-231). *Nature*. 1996;382(6587):180-182. doi: 10.1038/382180a0 [doi].

15. Stahl N, Borchelt DR, Hsiao K, Prusiner SB. Scrapie prion protein contains a phosphatidylinositol glycolipid. *Cell*. 1987;51(2):229-240. doi: 0092-8674(87)90150-4 [pii].

16. Moore RA, Taubner LM, Priola SA. Prion protein misfolding and disease. *Curr Opin Struct Biol*. 2009;19(1):14-22. doi: 10.1016/j.sbi.2008.12.007 [doi].

17. Aguzzi A, Calella AM. Prions: Protein aggregation and infectious diseases. *Physiol Rev*. 2009;89(4):1105-1152. doi: 10.1152/physrev.00006.2009 [doi].

18. Taylor DM. Inactivation of transmissible degenerative encephalopathy agents: A review. *Vet J*. 2000;159(1):10-17. doi: 10.1053/tvjl.1999.0406 [doi].

19. Bueler H, Aguzzi A, Sailer A, et al. Mice devoid of PrP are resistant to scrapie. *Cell*. 1993;73(7):1339-1347. doi: 0092-8674(93)90360-3 [pii].

20. Weissmann C. The state of the prion. *Nat Rev Microbiol*. 2004;2(11):861-871. doi: nrmicro1025 [pii].

21. Rigter A, Bossers A. Sheep scrapie susceptibility-linked polymorphisms do not modulate the initial binding of cellular to disease-associated prion protein prior to conversion. *J Gen Virol*. 2005;86(Pt 9):2627-2634. doi: 86/9/2627 [pii].

22. Priola SA. Prion protein and species barriers in the transmissible spongiform encephalopathies. *Biomed Pharmacother*. 1999;53(1):27-33.

23. Caughey B. Prion protein conversions: Insight into mechanisms, TSE transmission barriers and strains. *Br Med Bull*. 2003;66:109-120.

24. Kurt TD, Jiang L, Bett C, Eisenberg D, Sigurdson CJ. A proposed mechanism for the promotion of prion conversion involving a strictly conserved tyrosine residue in the beta2-alpha2 loop of PrPC. *J Biol Chem*. 2014;289(15):10660-10667. doi: 10.1074/jbc.M114.549030 [doi].

25. Haik S, Marcon G, Mallet A, et al. Doxycycline in creutzfeldt-jakob disease: A phase 2, randomised, double-blind, placebo-controlled trial. *Lancet Neurol*. 2014;13(2):150-158. doi: 10.1016/S1474-4422(13)70307-7 [doi].

26. Haik S, Brandel JP, Salomon D, et al. Compassionate use of quinacrine in creutzfeldt-jakob disease fails to show significant effects. *Neurology*. 2004;63(12):2413-2415. doi: 63/12/2413 [pii].

27. Whittle IR, Knight RS, Will RG. Unsuccessful intraventricular pentosan polysulphate treatment of variant creutzfeldt-jakob disease. *Acta Neurochir (Wien)*. 2006;148(6):677-9; discussion 679. doi: 10.1007/s00701-006-0772-y [doi].

28. Haik S, Brandel JP, Salomon D, et al. Compassionate use of quinacrine in creutzfeldt-jakob disease fails to show significant effects. *Neurology*. 2004;63(12):2413-2415. doi: 63/12/2413 [pii].

29. Trevitt CR, Collinge J. A systematic review of prion therapeutics in experimental models. *Brain*. 2006;129(Pt 9):2241-2265. doi: awl150 [pii].

30. Li L, Napper S, Cashman NR. Immunotherapy for prion diseases: Opportunities and obstacles. *Immunotherapy*. 2010;2(2):269-282. doi: 10.2217/imt.10.3 [doi].

31. Halliday M, Mallucci GR. Targeting the unfolded protein response in neurodegeneration: A new approach to therapy. *Neuropharmacology*. 2014;76 Pt A:169-174. doi: 10.1016/j.neuropharm.2013.08.034 [doi].

32. Soto C. Unfolding the role of protein misfolding in neurodegenerative diseases. *Nat Rev Neurosci*. 2003;4(1):49-60. doi: 10.1038/nrn1007 [doi].

33. Pfeifer A, Eigenbrod S, Al-Khadra S, et al. Lentivector-mediated RNAi efficiently suppresses prion protein and prolongs survival of scrapie-infected mice. *J Clin Invest*. 2006;116(12):3204-3210. doi: 10.1172/JCI29236 [doi].

34. Priola SA, Caughey B, Race RE, Chesebro B. Heterologous PrP molecules interfere with accumulation of protease-resistant PrP in scrapie-infected murine neuroblastoma cells. *J Virol*. 1994;68(8):4873-4878.

35. Vorberg I, Groschup MH, Pfaff E, Priola SA. Multiple amino acid residues within the rabbit prion protein inhibit formation of its abnormal isoform. *J Virol*. 2003;77(3):2003-2009.

36. Prusiner SB, Scott M, Foster D, et al. Transgenetic studies implicate interactions between homologous PrP isoforms in scrapie prion replication. *Cell*. 1990;63(4):673-686.

37. Telling GC, Scott M, Hsiao KK, et al. Transmission of creutzfeldt-jakob disease from humans to transgenic mice expressing chimeric human-mouse prion protein. *Proc Natl Acad Sci U S A*. 1994;91(21):9936-9940.

38. Telling GC, Scott M, Mastrianni J, et al. Prion propagation in mice expressing human and chimeric PrP transgenes implicates the interaction of cellular PrP with another protein. *Cell*. 1995;83(1):79-90.

39. Kimberlin RH. Experimental scrapie in the mouse: A review of an important model disease. *Sci Prog*. 1976;63(252):461-481.

40. Atarashi R, Moore RA, Sim VL, et al. Ultrasensitive detection of scrapie prion protein using seeded conversion of recombinant prion protein. *Nat Methods*. 2007;4(8):645-650. doi: 10.1038/nmeth1066.

41. Vorberg I, Raines A, Priola SA. Acute formation of protease-resistant prion protein does not always lead to persistent scrapie infection in vitro. *J Biol Chem*. 2004;279(28):29218-29225. doi: 10.1074/jbc.M402576200.

42. Paylor R, Nguyen M, Crawley JN, Patrick J, Beaudet A, Orr-Urtreger A. Alpha7 nicotinic receptor subunits are not necessary for hippocampal-dependent learning or sensorimotor gating: A behavioral characterization of Acra7-deficient mice. *Learn Mem*. 1998;5(4-5):302-316.

43. Harrington DP, Fleming TR. A class of rank test procedures for censored survival data. *Biometrika*. 1982;69:553-553-566.

44. Csiza CK, McMartin DN. Apparent acaridal dermatitis in a C57BL/6 nya mouse colony. *Lab Anim Sci*. 1976;26(5):781-787.

45. Caughey B, Kocisko DA, Raymond GJ, Lansbury PT,Jr. Aggregates of scrapie-associated prion protein induce the cell-free conversion of protease-sensitive prion protein to the protease-resistant state. *Chem Biol*. 1995;2(12):807-817.

46. Collinge J, Clarke AR. A general model of prion strains and their pathogenicity. *Science*. 2007;318(5852):930-936. doi: 318/5852/930 [pii].

47. Horiuchi M, Priola SA, Chabry J, Caughey B. Interactions between heterologous forms of prion protein: Binding, inhibition of conversion, and species barriers. *Proc Natl Acad Sci U S A*. 2000;97(11):5836-5841. doi: 10.1073/pnas.110523897 [doi].

48. Toupet K, Compan V, Crozet C, et al. Effective gene therapy in a mouse model of prion diseases. *PLoS One*. 2008;3(7):e2773. doi: 10.1371/journal.pone.0002773 [doi].
